# Supplementary material for: Human Papilloma Virus Vaccination in Patients with Rheumatic Diseases in France: A Study of Vaccination Coverage and Drivers of Vaccination
Source: J Clin Med. 2022 Jul 16;11(14):4137. doi: 10.3390/jcm11144137 (PMC9317620; doi:10.3390/jcm11144137)
Supplement: Supplementary file 1 [file jcm-11-04137-s001.zip › jcm-1797465-supplementary.pdf]

Supplementary Table S1. Vaccination schedule at the time of the study

Type of vaccine	Initial Vaccination	Catch-up program
Quadrivalent (Gardasil ®)	between 11 and 13 years old with 2 doses at 6-month intervals	between 14 and 19 years old with 3 doses administered according to a 0-, 2- and 6-month schedule
Bivalent (Cervarix ®)	between 11 and 14 years old with 2 doses at 6-month intervals	between 15 and 19 years old with 3 doses administered according to a 0-, 2- and 6-month schedule
Nonvalent (Gardasil9®)	between 11 and 14 years old with 2 doses at 6- to 13-month intervals	between 15 and 19 years old with 3 doses administered according to a 0-, 2- and 6-month schedule

Supplementary Table S1:
HPV vaccination schedule in France in 2018 according to the weekly epidemiological bulletin *(Bulletin Epidémiologie Hebdomadaire*) [13]

Supplementary Table S2. Comparison of vaccinated and non-vaccinated patients.

Characteristics	Vaccinated patients (n=28)	Non-vaccinated patients (n=43)	OR	95% CI	p-value*
**Bivariate Model**					
Age Group [11,12]	1 (4%)	22 (71%)	1		<0.001
Age Group [13,18]	22 (96%)	9 (29%)	53.68	5.85 – 492.29	
Hepatitis B not-vaccinated	5 (22%)	18 (58%)	1		0.040
Hepatitis B vaccinated	18 (78%)	13 (42%)	4.97	1.03 – 24.01	
**Bivariate Model**					
Paris	15 (65%)	11 (34%)	1		0.063
Lyon	8 (35%)	21 (66%)	0.33	0.10 – 1.08	
Hepatitis B not-vaccinated	5 (22%)	18 (56%)	1		0.024
Hepatitis B vaccinated	18 (78%)	14 (44%)	4.01	1.15 – 14.00	

* Likelihood ratio test
